# Supplementary material for: Dual Optoelectronic Organic Field-Effect Device: Combination of Electroluminescence and Photosensitivity
Source: Molecules. 2024 May 28;29(11):2533. doi: 10.3390/molecules29112533 (PMC11173939; doi:10.3390/molecules29112533)
Supplement: Supplementary file 1 [file molecules-29-02533-s001.zip › molecules-2976810-supplementary.pdf]

Dual Optoelectronic Organic Field-Effect Device: Combination of Electroluminescence and Photosensitivity

SUPPORTING INFORMATION

Crystal images

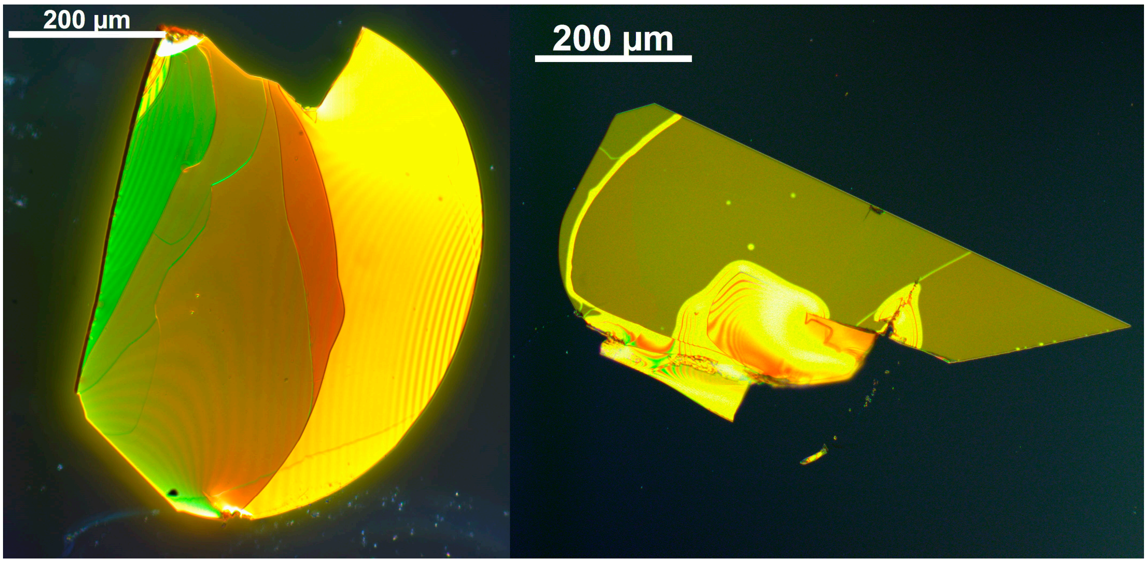

Figure S1. Microscopy images of vapor-grown TMS-P4TP-TMS single crystals. The images were captured with the use of differential interference-contrast microscopy in circularly polarized light (AxioImager A2m, Zeiss with 10x objective).

X-ray data

Figure S2 illustrates the molecular geometries of four independent molecules (A, B, C, D) in the unit cell of corresponding TMS-P4TP-TMS crystal. All the molecules show moderate torsion angles (3–7°), and hence are almost flat, except for the one phenyl ring in each molecule (C1-C6 in A and B molecules and C23-C28 in C and D molecules), which is twisted relative to the rest five rings (13–26°). This is in line with the expected planar structure of this molecule, since its shorter analog TMS-PTTP-TMS has a planar structure in crystal.[1]

Crystal-growth morphology was calculated from the solved crystal structures using the Morphology module of the Materials Studio Package[2] (Compass II force field).[3]

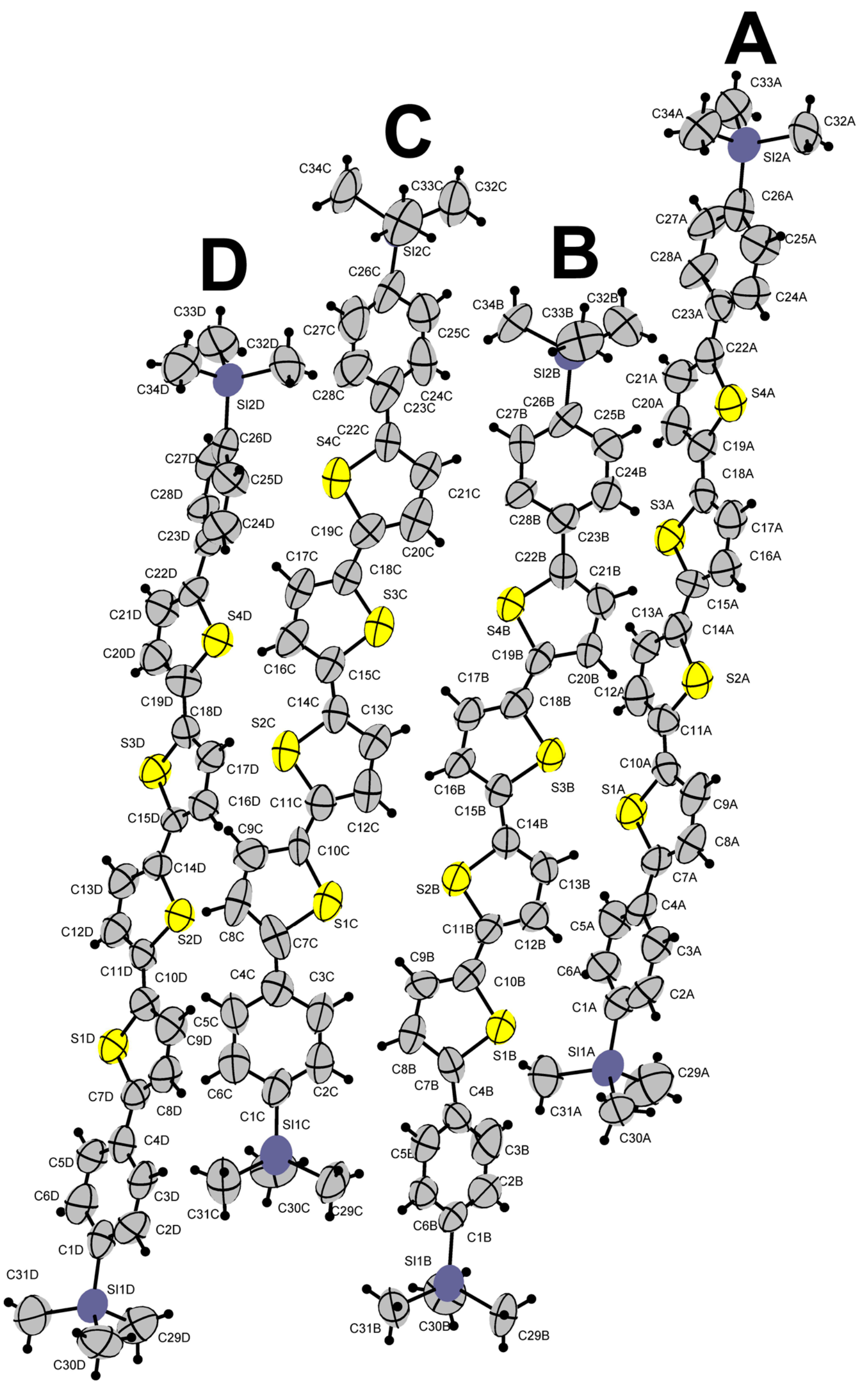

Figure S2. The designation of atoms in TMS-P4TP-TMS molecule. Atomic ellipsoids depict the positions of atoms with a 50% probability level. The relevant dihedral angles for A molecule of TMS-P4TP-TMS are: between adjacent phenylene and thiophene C1A-C6A/S1A-C10A – 13°, C23A-C28A/S4A-C22A – 3.8°; adjacent thiophene rings S1A-C10A/S2A-C14A – 5.6°, S2A-C14A/S3A-C18A – 1.3°, S3A-C18A/S4A-C22A – 4.2°. The relevant dihedral angles for B molecule of TMS-P4TP-TMS are: between adjacent phenylene and thiophene C1B-C6B/S1B-C10B – 32°, C23B-C28B/S4B-C22B – 5.3°; adjacent thiophene rings S1B-C10B/S2B-C14B – 2.7°, S2B-C14B/S3B-C18B – 10.7°, S3B-C18B/S4B-C22B – 2.3°. The relevant dihedral angles for C molecule of TMS-P4TP-TMS are: between adjacent phenylene and thiophene C1B-C6B/S1B-C10B – 2.4°, C23B-C28B/S4B-C22B – 28.8°; adjacent thiophene rings S1B-C10B/S2B-C14B – 1.4°, S2B-C14B/S3B-C18B – 5.8°, S3B-C18B/S4B-C22B – 4°. The relevant dihedral angles for D molecule of TMS-P4TP-TMS are: between adjacent phenylene and thiophene C1B-C6B/S1B-C10B – 2.2°, C23B-C28B/S4B-C22B – 28°; adjacent thiophene rings S1B-C10B/S2B-C14B – 5.3°, S2B-C14B/S3B-C18B – 7°, S3B-C18B/S4B-C22B – 5.3°. The

relevant torsion angles for A molecule are: C3A-C8A – 13.5°, C9A-S2A – -2.8°, C13A-S3A – 0.2°, C17A-S4A – -8°, C21A-C28A – -0.3°. The relevant torsion angles for B molecule are: C5B-C8B – -26°, C9B-S2B – 2.6°, C13B-S3B – 0.2°, C17B-S4B – 0.9°, C21B-C24B – -0.2°. The relevant torsion angles for C molecule are: C5C-C8C – 3.5°, C9C-S2C – 3.1°, C13C-S3C – 7°, C17C-S4C – -1.4°, C21C-C24C – 26°. The relevant torsion angles for D molecule are: C3A-C8A – -0.3°, C9A-S2A – 4.2°, C13A-S3A – -6.7°, C17A-S4A – 6.5°, C21A-C28A – -24.5°.

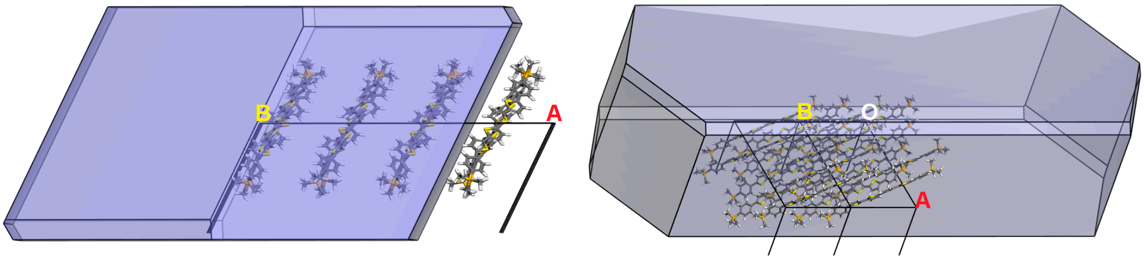

Figure S3. Calculated TMS-P4TP-TMS crystal growth morphology. The unit cell ***ab***-plane is parallel to the basal plane of the crystal habit.

Hirshfeld surface analysis

Hirshfeld surface analysis[4] of TMS-P4TP-TMS crystal structure was carried out to reveal the intermolecular interactions governing the packing motif in this crystal. The Hirshfeld surface defines the space occupied by a molecule in the crystal: inside this surface, the electron density from the given molecule is larger than that from the others.[5] Fig. S4 displays the Hirshfeld surfaces for the TMS-P4TP-TMS crystal mapped with normalized contact distance d_{norm}, curvedness (C) and electrostatic potential (ESP); see Ref.[6] their definitions. The curvedness maps shown in Fig. S4b can be used to analyze the molecular packing motifs.[5] Particularly, the absence of relatively large green flat regions on the curvedness map of TMS-P4TP-TMS indicates a herringbone packing. Fig. S4c shows the electrostatic potential of TMS-P4TP-TMS: the thiophene and phenylene rings have the electronegative center and the electropositive periphery (hydrogen atoms), which is typical for thiophene-phenylene co-oligomers.[7,8]

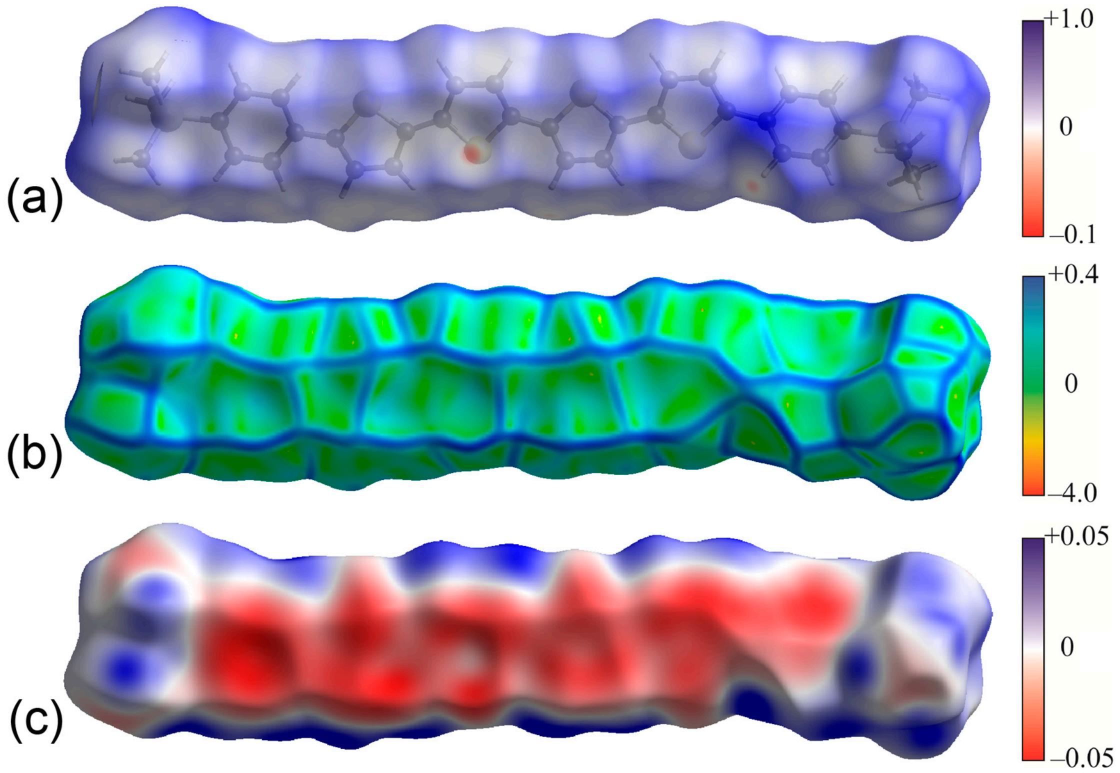

Figure S4. Hirshfeld surfaces of TMS-P4TP-TMS mapped with normalized contact distance d_{norm} (a), curvature C (b) and ESP (±65.6 kJ mol^{-1} per unit charge) (c). Red spots in (a) indicate intermolecular contacts closer than the sum of the van der Waals radii (close contacts), blue spots are referred to longer contacts, and contacts around the sum of van der Waals radii (moderate contacts) are white.

Optical data

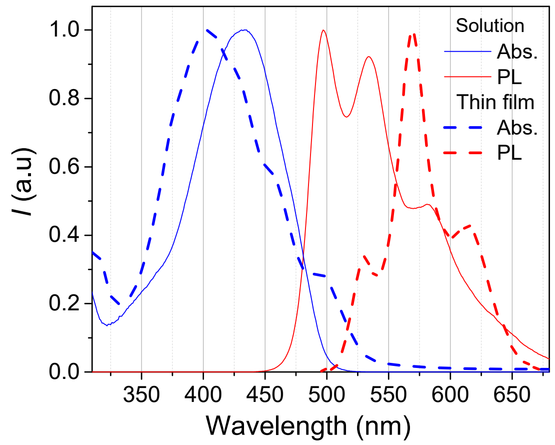

Figure S5. Absorption and photoluminescence spectra of TMS-P4TP-TMS in solution in tetrahydrofuran (THF) and in thin film.

Experimental setup for OFET characterization

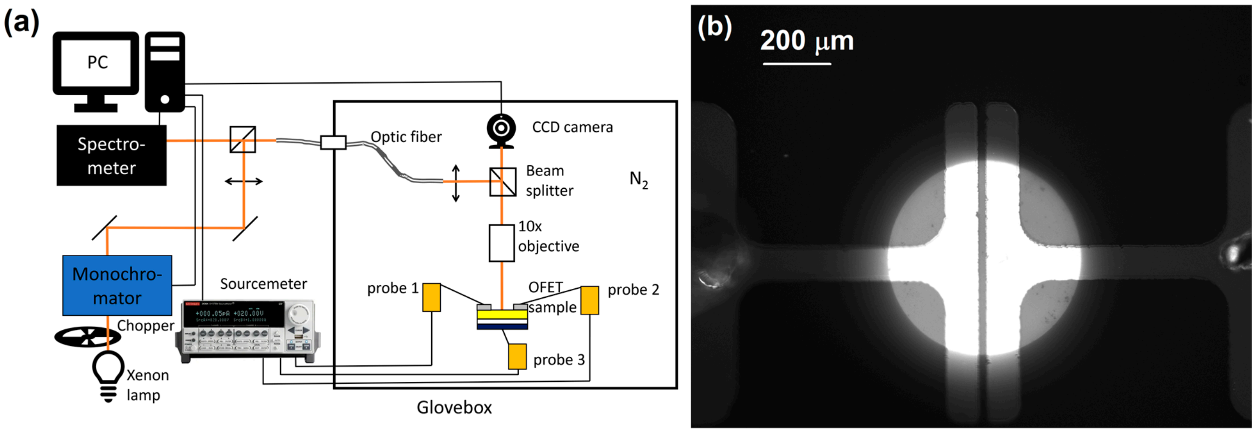

Figure S6. Scheme of experimental setup for OPT samples characterization (a), image of OPT sample under incident illumination, view from above (b).

Optical and atomic force microscopy data

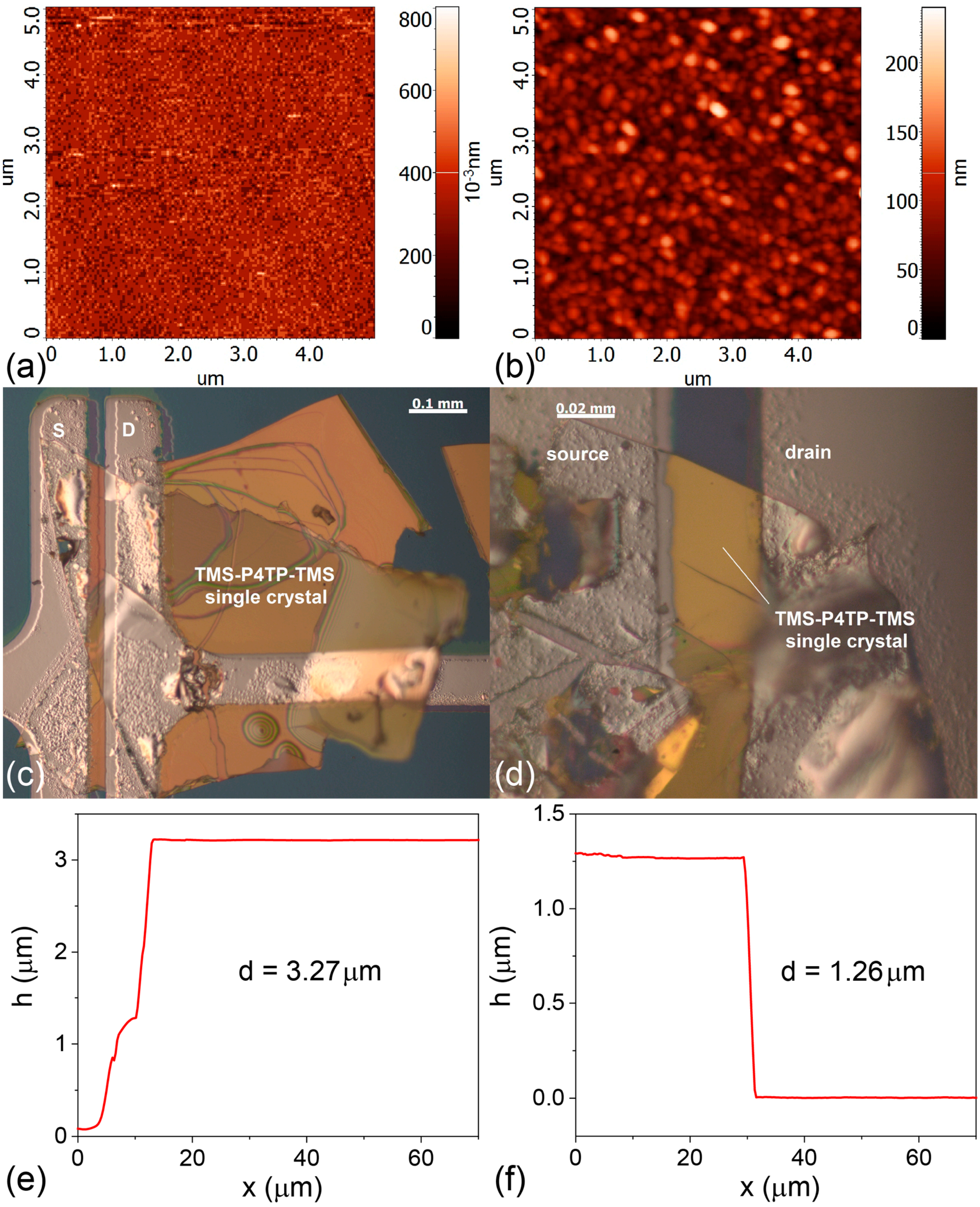

Figure S7. AFM maps for a 50-nm thin-film TMS-P4TP-TMS active layer (a) and for single crystal grown from this material (b). Optical microscopy images captured in the C-DIC regime of two OFET samples with single-crystalline active layers of two different thicknesses (c,d), and the AFM profiles near their edges (e,f).

Output characteristics

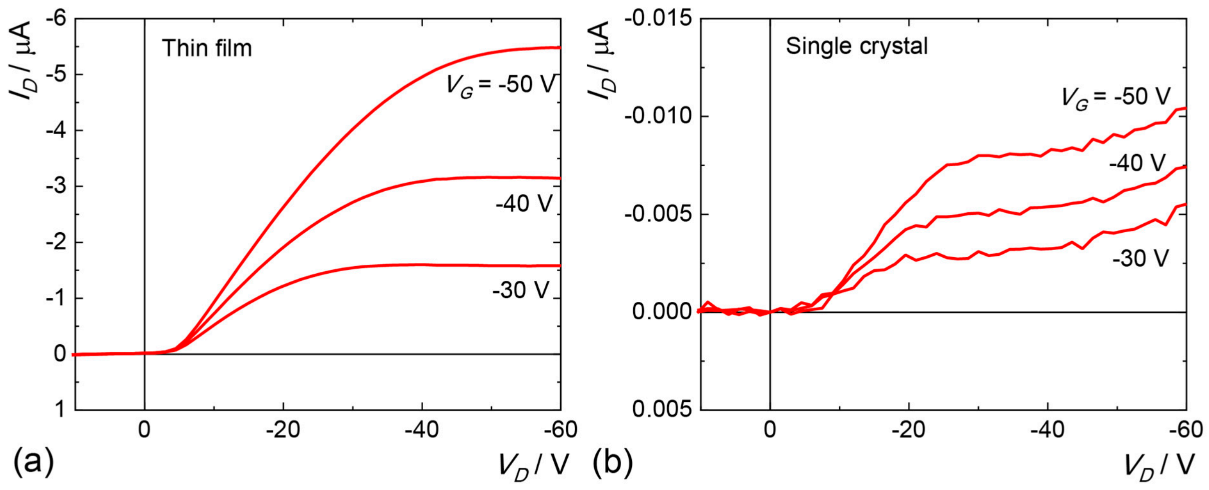

Figure S8. Output characteristics for thin-film (a) and single crystal (b) OFET.

Electroluminescence raw images

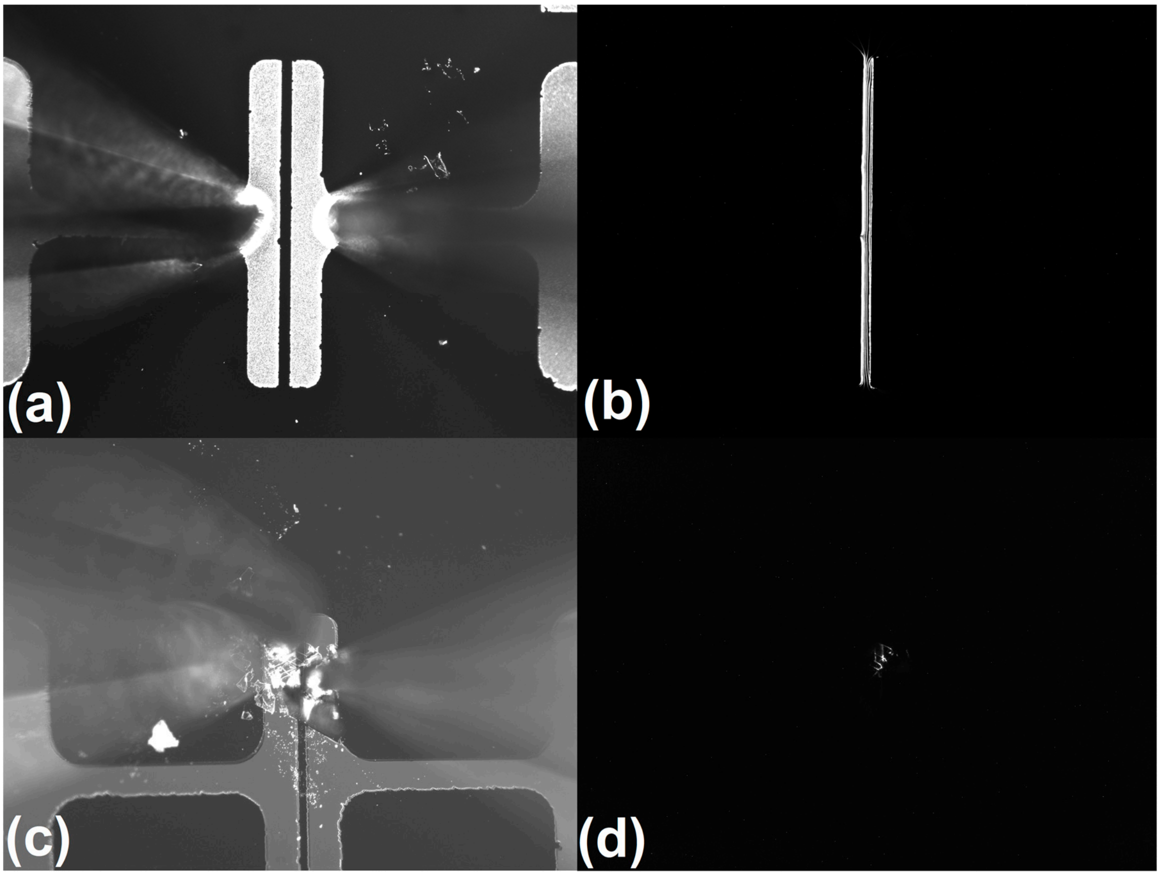

Figure S9. Original raw images of working OFETs based on thin film (a,b) and single crystal (c,d) under the backlight (a,c) and in dark (b,d). Images in dark were captured with long exposure of 20 s for thin film (b) and 60 s for single crystal (d) when the drain and gate voltages were applied to OFET and changed stepwise V_G from –60 to 30 V at V_D = –50 V

Photocurrent in single-crystal OFET

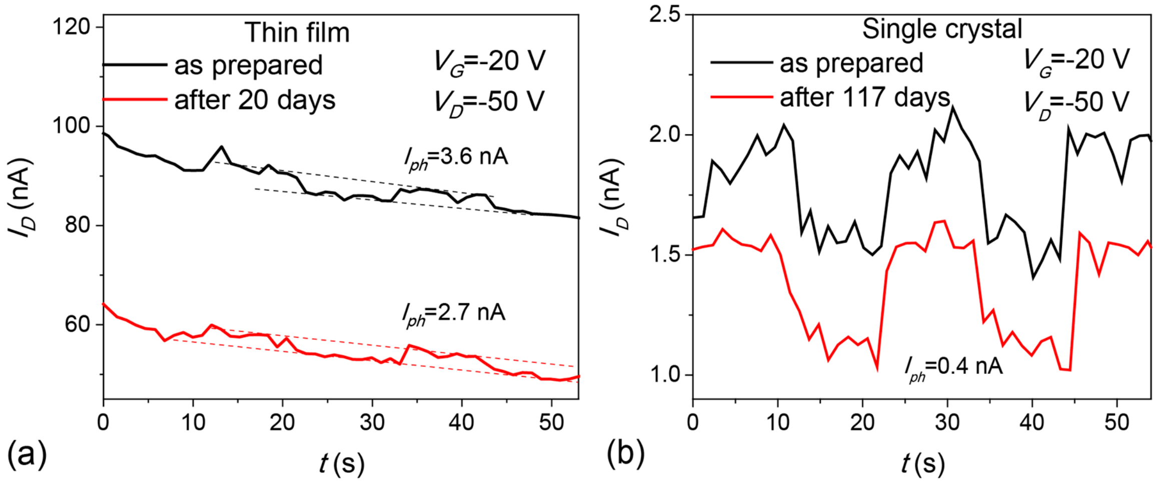

Figure S10. Drain current I_D dependence on time t for thin film (a) and single crystal (b) based OFET under modulated incident illumination with period of 20 s immediately after preparation and after 20 or 117 days storing in inert atmosphere; while dark current drops about 1.5 times, photocurrent remains almost unchanged and equal about 0.4 nA for single crystal based OFET, for thin-film based OFET photocurrent drops by quarter even after 20 days of storing in the same conditions as for single crystal based one.

References

1. Kudryashova, L.G.; Kazantsev, M.S.; Postnikov, V.A.; Bruevich, V.V.; Luponosov, Y.N.; Surin, N.M.; Borshchev, O.V.; Ponomarenko, S.A.; Pshenichnikov, M.S.; Paraschuk, D.Y. Highly Luminescent Solution-Grown Thiophene-Phenylene Co-Oligomer Single Crystals. *ACS Applied Materials & Interfaces* **2016**, *8*, 10088-10092, doi:10.1021/acsami.5b11967.
2. *Materials Studio package*, BIOVIA, Dassault Systèmes: San Diego, USA, 2017.
3. Sun, H.; Jin, Z.; Yang, C.; Akkermans, R.L.C.; Robertson, S.H.; Spenley, N.A.; Miller, S.; Todd, S.M. COMPASS II: extended coverage for polymer and drug-like molecule databases. *Journal of Molecular Modeling* **2016**, *22*, 47, doi:10.1007/s00894-016-2909-0.
4. Spackman, P.R.; Turner, M.J.; McKinnon, J.J.; Wolff, S.K.; Grimwood, D.J.; Jayatilaka, D.; Spackman, M.A. CrystalExplorer: a program for Hirshfeld surface analysis, visualization and quantitative analysis of molecular crystals. *Journal of Applied Crystallography* **2021**, *54*, 1006-1011, doi:10.1107/S1600576721002910.
5. Spackman, M.A.; Byrom, P.G. A novel definition of a molecule in a crystal. *Chemical Physics Letters* **1997**, *267*, 215-220, doi:10.1016/S0009-2614(97)00100-0.
6. McKinnon, J.J.; Spackman, M.A.; Mitchell, A.S. Novel tools for visualizing and exploring intermolecular interactions in molecular crystals. *Acta Crystallographica Section B* **2004**, *60*, 627-668, doi:10.1107/S0108768104020300.
7. Sosorev, A.Y.; Trukhanov, V.A.; Maslennikov, D.R.; Borshchev, O.V.; Polyakov, R.A.; Skorotetcky, M.S.; Surin, N.M.; Kazantsev, M.S.; Dominskiy, D.I.; Tafeenko, V.A.; et al. Fluorinated Thiophene-Phenylene Co-Oligomers for Optoelectronic Devices. *ACS Applied Materials & Interfaces* **2020**, *12*, 9507-9519, doi:10.1021/acsami.9b20295.
8. Trukhanov, V.A.; Dominskiy, D.I.; Parashchuk, O.D.; Feldman, E.V.; Surin, N.M.; Svidchenko, E.A.; Skorotetcky, M.S.; Borshchev, O.V.; Paraschuk, D.Y.; Sosorev, A.Y. Impact of N-substitution on structural, electronic, optical, and vibrational properties of a thiophene–phenylene co-oligomer. *RSC Advances* **2020**, *10*, 28128-28138, doi:10.1039/D0RA03343J.
